# Fluoxetine Induces Apoptosis through Extrinsic/Intrinsic Pathways and Inhibits ERK/NF-κB-Modulated Anti-Apoptotic and Invasive Potential in Hepatocellular Carcinoma Cells In Vitro

**DOI:** 10.3390/ijms20030757

**Published:** 2019-02-11

**Authors:** Wei-Ting Chen, Fei-Ting Hsu, Yu-Chang Liu, Cheng-Hsien Chen, Li-Cho Hsu, Song-Shei Lin

**Affiliations:** 1 Department of Medical Imaging and Radiological Sciences, Central Taiwan University of Science and Technology, Taichung 406, Taiwan; wt820368@yahoo.com.tw (W.-T.C.); kevinyc.liu@gmail.com (Y.-C.L.); 2 Department of Psychiatry, Zuoying Branch of Kaohsiung Armed Forces General Hospital, Kaohsiung 813, Taiwan; 3 Department of Physical Therapy, Shu-Zen Junior College of Medicine and Management, Kaohsiung 821, Taiwan; 4 Department of Biological Science and Technology, China Medical University, Taichung 404, Taiwan; sakiro920@mail.cmu.edu.tw; 5 Department of Radiation Oncology, Chang Bing Show Chwan Memorial Hospital, Changhua 505, Taiwan; 6 Department of Radiation Oncology, Show Chwan Memorial Hospital, Changhua 500, Taiwan; 7 Department of Surgery, Show Chwan Memorial Hospital, Changhua 500, Taiwan; 8 Division of Endocrinology and Metabolism, Department of Medicine, National Yang-Ming University Hospital, Yilan 260, Taiwan

**Keywords:** fluoxetine, human hepatocellular carcinoma, NF-κB, apoptosis

## Abstract

The aim of the present study was to verify the effects of fluoxetine on dysregulation of apoptosis and invasive potential in human hepatocellular carcinoma (HCC) SK-Hep1 and Hep3B cells. Cells were treated with different concentrations of fluoxetine for different times. MTT (3-(4,5-Dimethylthiazol-2-yl)-2,5-Diphenyltetrazolium Bromide) assays were used for testing the effects of fluoxetine on cell viability. The regulation of apoptosis signaling, and anti-apoptotic, proliferation, and metastasis-associated proteins after fluoxetine treatment were assayed by flow cytometry and Western blotting assay. The detection of nuclear factor kappa-light-chain-enhancer of activated B cells (NF-κB) activation after fluoxetine treatment was performed by NF-κB reporter gene assay. The results demonstrated that fluoxetine significantly reduced cell viability, cell migration/invasion, NF-κB, extracellular signal-regulated kinases (ERK) activation, and expression of anti-apoptotic (Cellular FLICE (FADD-like IL-1β-converting enzyme)-inhibitory protein (C-FLIP), Myeloid cell leukemia-1 (MCL-1), X-Linked inhibitor of apoptosis protein (XAIP), and Survivin), proliferation (Cyclin-D1), angiogenesis (vascular endothelial growth factor (VEGF)), and metastasis-associated proteins (matrix metalloproteinase-9 (MMP-9)). Fluoxetine also significantly induced apoptosis, unregulated extrinsic (activation of first apoptosis signal protein and ligand (Fas/FasL), and caspase-8) and intrinsic (loss of mitochondrial membrane potential (ΔΨm) pathways and increased Bcl-2 homologous antagonist killer (BAK) apoptosis signaling. Taken together, these results demonstrated that fluoxetine induced apoptosis through extrinsic/intrinsic pathways and diminished ERK/NF-κB-modulated anti-apoptotic and invasive potential in HCC cells in vitro.

## 1. Introduction

Hepatocellular carcinoma (HCC), the most common primary malignancy of the liver, is the second leading cause of cancer-related death worldwide [1]. Dysregulation of apoptosis and high metastatic ability is associated with poor prognosis in patients with HCC [2,3]. Sorafenib and regorafenib are oral multi-kinase inhibitors which promote apoptosis and suppress anti-apoptotic and metastatic activity and are approved for treatment of HCC [4,5]. Therefore, induction of apoptosis and inhibition of anti-apoptotic and metastatic activity may be an available treatment strategy for patients with HCC.

Antidepressants are used to treat depression and improve quality of life in cancer patients [6]. In addition, the anti-cancer effects of antidepressants have been found in several cancer cells [7,8]. Antidepressants modulate inhibition of tumor growth through triggering antitumor immunity, inducing apoptosis, and blocking intracellular signaling transduction [9,10]. Many antidepressants such as desipramine, sertraline, and fluoxetine, have been shown to induce apoptosis in HCC [11,12,13].

Fluoxetine, a selective serotonin reuptake inhibitor (SSRI) antidepressant, is used for the treatment of depression and anxiety disorders [14]. In ovarian cancer-related studies, the apoptotic effect of fluoxetine on human epithelial ovarian cancer cell lines OVCAR-3 and SK-OV-3 growth which was regulated by mitochondria-mediated cell death process was found [15]. In Stepulak et al.’s study, they also suggested that fluoxetine inhibits the extracellular signal-regulated kinase pathway and suppresses growth of colon cancer cells and lung cancer cells [16]. Fluoxetine may also reverse breast cancer cells’ resistance and enhance the chemosensitivity to common chemotherapy drugs, such as adriamycin and paclitaxel [17]. Mun et al. reported that fluoxetine induces apoptosis via depletion of the mitochondrial membrane potential (ΔΨ_m_), the formation of reactive oxygen species, and the inhibition of mitogen-activated protein kinase (MAPK) activation in HCC Hep3B cells [13]. Whether fluoxetine inhibits anti-apoptotic and metastatic activity in HCC remains ambiguous. Therefore, we investigated the effect of fluoxetine on the dysregulation of apoptosis and invasive potential in HCC.

## 2. Results

### 2.1. Fluoxetine Decreased the Cell Viability and NF-κB Activation of Sk-Hep1 and Hep3B Cells

**Figure 1:**
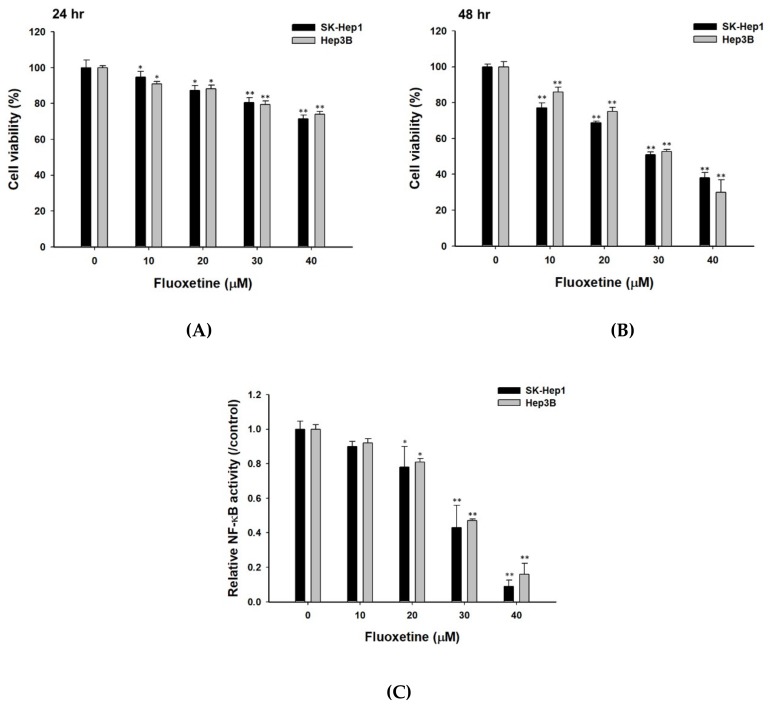
Fluoxetine reduced cell viability and NF-κB activation in SK-Hep1 and Hep3B cells. Different concentrations of fluoxetine (0, 10, 20, 30, 40 µM) were given to SK-Hep1 and Hep3B cells for 24 and 48 h and then cell viability was evaluated by MTT assay of two different hepatocellular carcinoma (HCC cells) at: (**A**) 24 h, and (**B**) 48 h. (**C**) A NF-κB reporter gene assay was performed on SK-Hep1/*NF-κB-luc2* and Hep3B/*NF-κB-luc2* cells at 48 h. * *p* < 0.05 and ** *p* < 0.01, significant difference between fluoxetine-treated groups and the control as analyzed by Student’s t test.

SK-Hep1 and Hep3B cells were treated with 0–40 µM of fluoxetine for 24 and 48 h. Cells were examined for the percentage of viable cells and NF-κB activation by MTT and NF-κB reporter gene assay. The results indicated that fluoxetine significantly decreased the quantity of viable cells (Figure 1A). The cell viability of SK-Hep1 and Hep3B cells was reduced with an increase of fluoxetine concentration and incubation time. Cell viability was inhibited by 50–60% as compared to the control group (vehicle treatment) at 48 h after 30 µM and 40 µM fluoxetine treatment. Therefore, both 30 µM and 40µM doses of fluoxetine were selected for subsequent experiments. Fluoxetine significantly reduced relative NF-κB activation in SK-Hep1 and Hep3B cells by 20–80% as compared to the control group (Figure 1B). 

### 2.2. Fluoxetine Induced Apoptosis and Reduced Expression of Anti-Apoptotic Proteins in SK-Hep1 Cells

**Figure 2:**
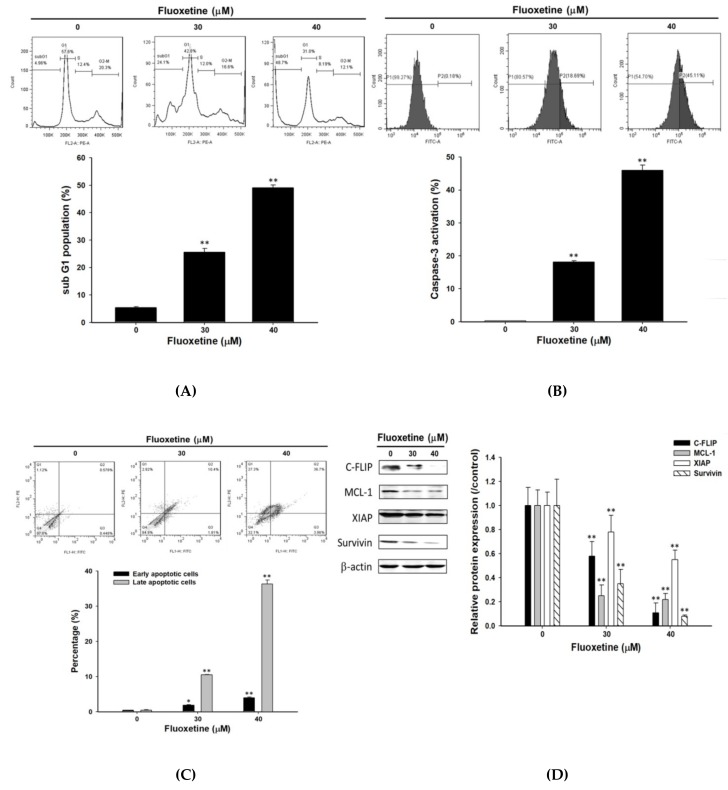
Fluoxetine induced apoptosis and inhibited expression of anti-apoptotic proteins in SK-Hep1 cells. Cells were treated with different concentrations (0, 30, and 40 µM) of fluoxetine for 48 h, respectively. The effect of fluoxetine on dysregulation of apoptosis in SK-Hep1 cells was evaluated with flow cytometry and western blotting. (**A**) Cell cycle analysis; (**B**) detection of caspase-3 activation; (**C**) evaluation of early and late apoptosis events by Annexin V/PI-double staining; (**D**) expression of anti-apoptotic proteins (C-FLIP, MCL-1, XIAP, and Survivin) are presented with Western blotting assay. Quantification data were averaged over three repeated experiments. * *p* < 0.05 and ** *p* < 0.01, significant difference between the control and fluoxetine-treated groups.

Detection of cell cycle and caspase-3 activation, Annexin V/PI-double staining, and western blotting were used to investigate the effect of fluoxetine on dysregulation of apoptosis in SK-Hep1 cells. In Figure 2A,B indicated fluoxetine significantly induced accumulation of sub-G_1_ and caspase-3 activation by 25–50% and 18–48%. The results of dot plots (Figure 2C) indicated that 30 µM and 40 µM of fluoxetine induced apoptosis of cells, with an increase in the percentage of early apoptotic cells (2–4%) and late apoptotic cells (10–30%). Fluoxetine significantly induced early-stage and late-stage apoptosis in a dose-dependent manner. Expression of anti-apoptotic proteins (C-FLIP, MCL-1, XIAP, and Survivin) was reduced with fluoxetine treatment by 22–92% as compared to the control group (Figure 2D).

### 2.3. Fluoxetine Promoted Extrinsic and Intrinsic Apoptotic Signaling Transduction in SK-Hep1 and Hep3B Cells

**Figure 3:**
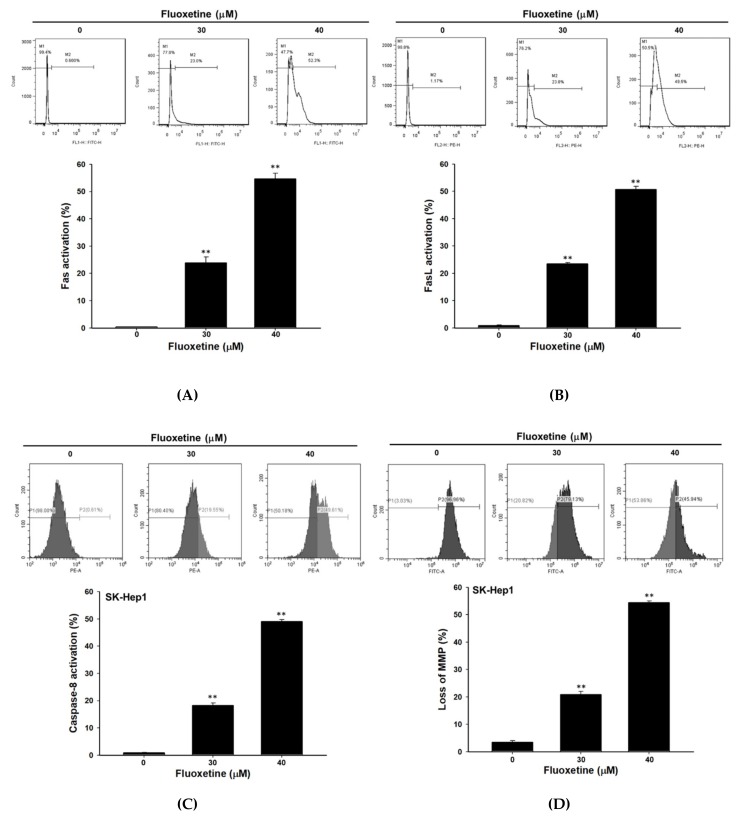

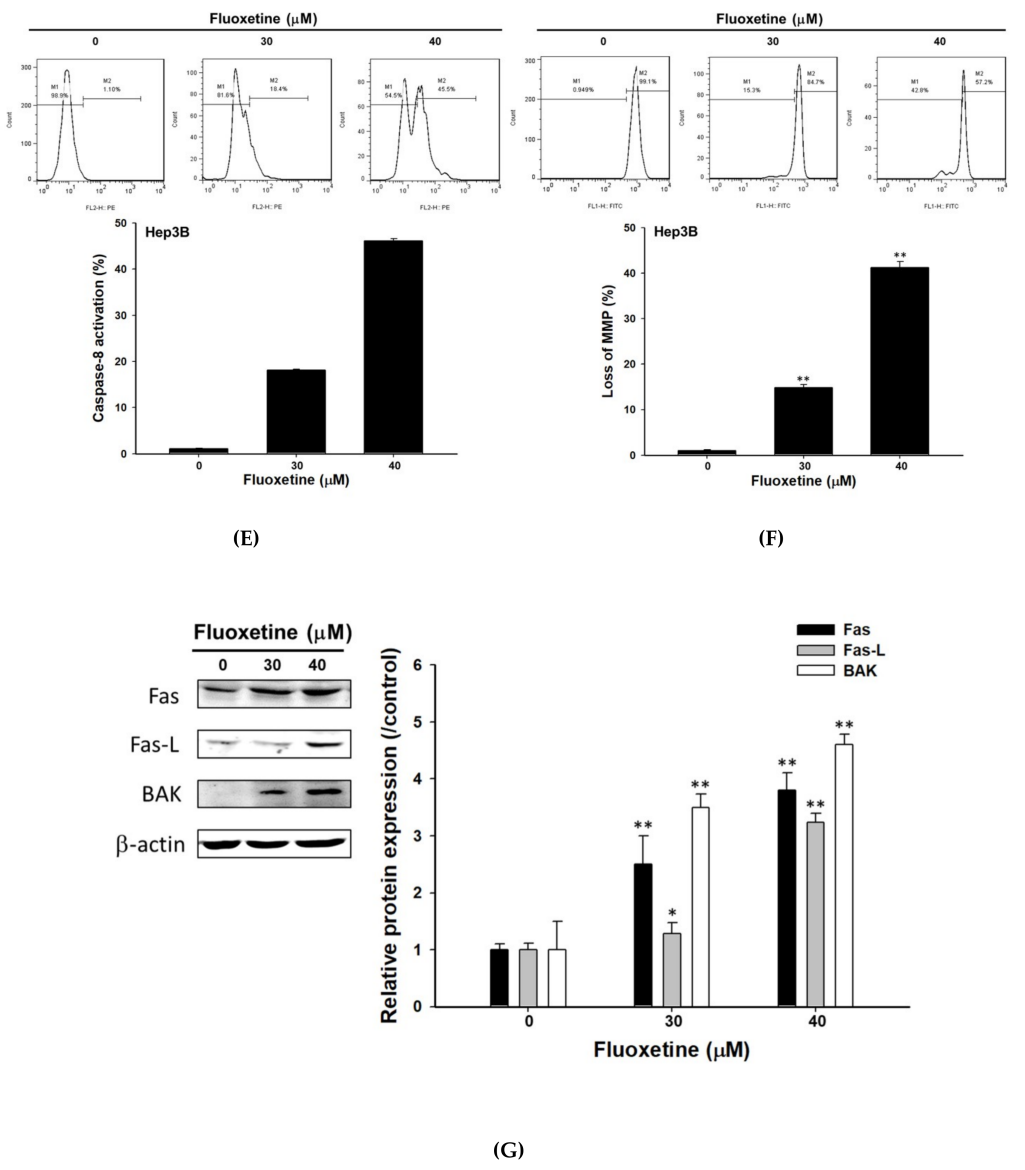
Fluoxetine modulated extrinsic and intrinsic apoptosis pathways in SK-Hep1 and Hep3B cells. Cells were treated with different concentrations (0, 30, and 40 µM) of fluoxetine for 48 h, respectively. Extrinsic and intrinsic apoptotic signaling was determined by flow cytometry and western blotting assay. Activation of (**A**) Fas, (**B**) FasL, and (**C**) caspase-8 was determined on SK-Hep1 cells with flow cytometry. (**D**) Detection of ΔΨ_m_ on SK-Hep1 cells by flow cytometry. (**E**) Detection of caspase-8 activation on Hep3B cells. (**F**) Detection of ΔΨ_m_ on Hep3B cells. (**G**) Protein levels of Fas, FasL, and BAK on SK-Hep1 cells were investigated with Western blotting assay. Quantification data were normalized by β-actin expression and averaged over three repeated experiments. * *p* < 0.05, ** *p* < 0.01, significant difference between control and fluoxetine-treated groups.

To investigate apoptosis signaling induced by fluoxetine, we performed various apoptosis determination methods as follows. The results shown in Figure 3A–C revealed that fluoxetine promoted the activation of Fas, FasL, and caspase-8. Loss of mitochondria membrane potential (ΔΨ_m_) is required for intrinsic apoptosis. Figure 3D indicated fluoxetine significantly triggered loss of ΔΨ_m_. Additionally, we found extrinsic and intrinsic apoptosis mechanisms were both activated by fluoxetine in Hep3B cells as well (Figure 3E,F). Protein levels of Fas, FasL, and BAK were significantly enhanced by fluoxetine treatment in SK-Hep1 cells (Figure 3G).

### 2.4. Fluoxetine Suppressed Cell Migration/Invasion and Reduced ERK Activation and Expression of Metastasis-Associated and Proliferative Proteins in SK-Hep1 and Hep3B Cells

**Figure 4:**
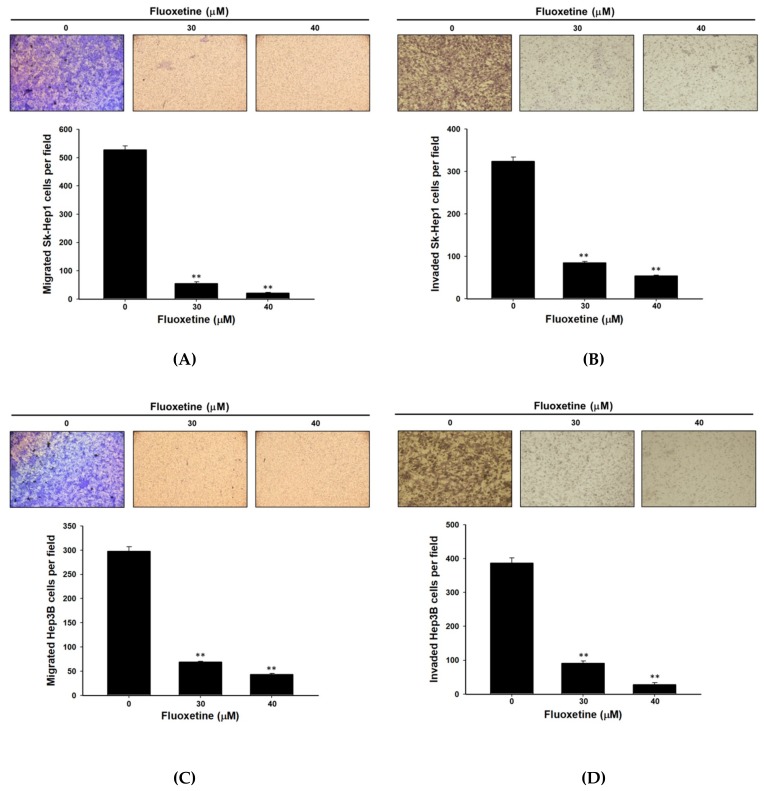

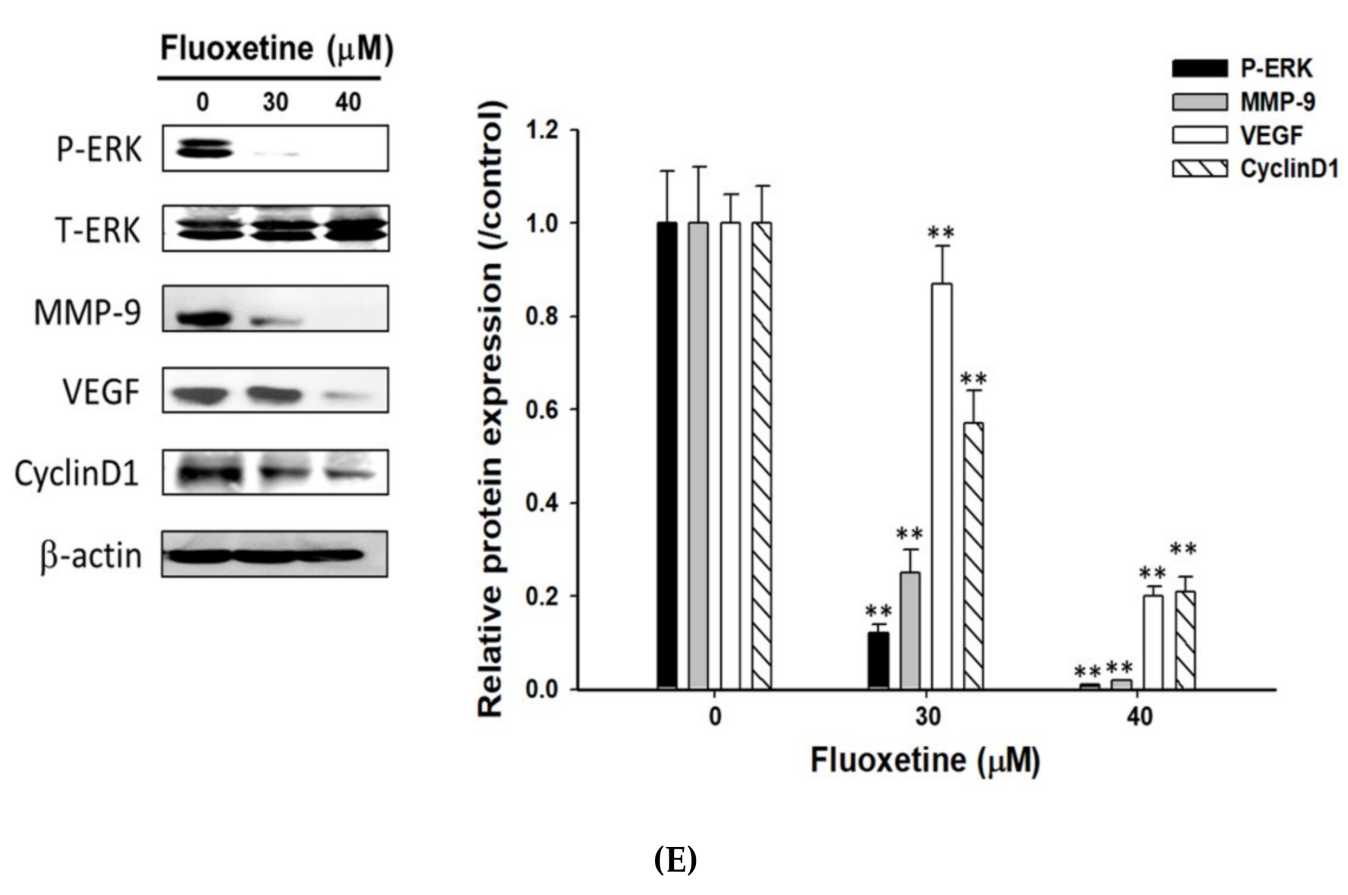
Fluoxetine decreased the cell migration/invasion and expression of pERK, metastasis-associated and proliferative proteins in SK-Hep1 and Hep3B cells. SK-Hep1 and Hep3B cells were treated with 0, 30 µM, and 40 µM of fluoxetine for 48 h. Then (**A**,**C**) migration assay or (**B**,**D**) invasion assay was performed. Quantification results were measured by four selected field per group. (**E**) Western blots were performed with P-ERK, T-ERK, MMP-9, VEGF and CyclinD1 antibodies to the indicated protein expression after fluoxetine treatment on SK-Hep1 cells. Quantification data were normalized by β-actin expression and averaged over three repeated experiments. ** *p* < 0.01, significant difference between control and fluoxetine-treated groups.

Transwell cell migration and invasion assays were used for measuring cell migration and invasion in SK-Hep1 and Hep3B cells after exposure to fluoxetine. The results indicated that fluoxetine significantly inhibited cell migration and invasion by 80–90% and 70–80%, respectively, as compared to the control group (Figure 4A,B). Furthermore, fluoxetine may also decrease the number of migration and invasion Hep3B cells (Figure 4C,D). As shown in Figure 4E, fluoxetine significantly reduced levels of metastasis-associated (MMP-9 and VEGF) and proliferative proteins (Cyclin-D1). Moreover, we also investigated effect of fluoxetine on MAPK/ERK activation with western blotting. The results indicated that fluoxetine markedly suppressed the protein level of pERK (Figure 4E).

### 2.5. Fluoxetine Not Only Induced Apoptosis, but Suppressed Tumor Progression in SK-Hep1 and Hep3B Cells

**Figure 5:**
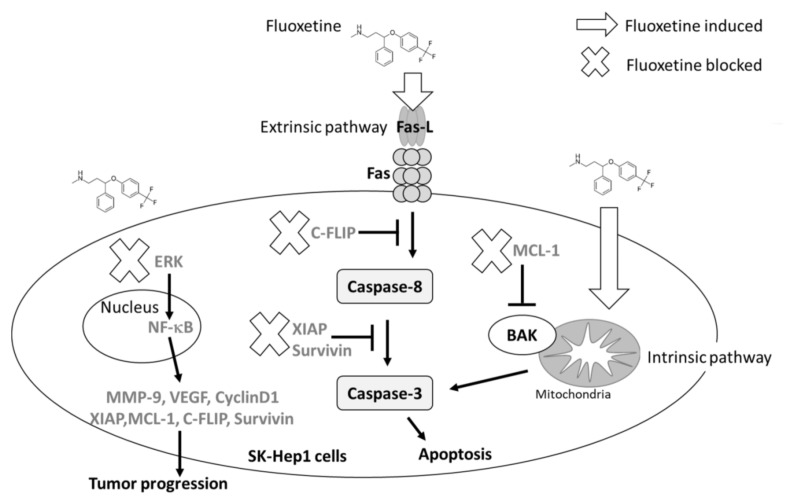
The potential anti-HCC properties of fluoxetine. Fluoxetine induces apoptosis through extrinsic/intrinsic pathways and inhibits ERK/NF-κB-modulated anti-apoptotic and metastatic activity in SK-Hep1 and Hep3B cells.

To explain the mechanism of fluoxetine on Sk-Hep1 and Hep3B cells, we propose the mechanism shown in Figure 5 based on our experimental results. Fluoxetine may trigger extrinsic (through death receptor) and intrinsic (through mitochondria) apoptosis mechanisms. Fluoxetine also reduced the production of anti-apoptosis proteins such as C-FLIP, MCL-1 and XIAP. Moreover, tumor invasion and migration ability were both suppressed by fluoxetine. Tumor invasion-related protein MMP-9 was significantly inhibited by fluoxetine. ERK-mediated tumor progression signaling was also blocked by fluoxetine. In sum, fluoxetine may enhance apoptosis effects and suppress tumor progression of HCC.

## 3. Discussion

Initiation of the extrinsic apoptotic pathway is mediated by cell death receptor Fas/Fas ligand (FasL) interaction [18]. Resistance to apoptosis is associated with upregulation of anti-apoptotic proteins and downregulation of pro-apoptotic proteins in HCC [2]. Reduction of Fas expression was found in HCC with poor differentiation [19]. Overexpression of anti-apoptotic proteins, including MCL-1, C-FLIP, XIAP, and Survivin, is implicated with a poor prognosis in patients with HCC [20,21]. MCL-1 and C-FLIP mediate resistance to apoptosis through suppression of intrinsic and extrinsic apoptotic signaling transduction. Both XIAP and Survivin, diminish apoptosis induced by anti-cancer agents through a decrease of caspase-3 activation [20,22,23]. Pro-apoptotic protein BAK-mediated dissipation of the mitochondrial membrane potential (ΔΨ_m_) is blocked by MCL-1 [24]. Furthermore, Fluoxetine has been demonstrated to induce cell death through induction of the mitochondrial apoptosis pathway in HCC Hep3B cells [13]. In our data, we found that fluoxetine significantly induced apoptosis, enhanced expression of apoptotic proteins (Fas, FasL, and BAK), and triggered extrinsic and intrinsic apoptotic signaling (increases of Fas, FasL, Caspase-8 activation and induced the loss of ΔΨm). In addition, expression of anti-apoptotic proteins (C-FLIP, XIAP, MCL-1, and Survivin) was significantly reduced by fluoxetine treatment.

Cyclin-D1, an initiator of cell cycle progression in actively proliferating cells, is overexpressed and correlated with unfavorable survival in HCC [25,26]. Vascular endothelial growth factor (VEGF), an angiogenic factor, induces the formation of blood vessels which is required for tumor growth and metastasis. Matrix metalloproteinase-9 (MMP-9), gelatinase B, enhances tumor invasion and metastasis through degradation of the basement membrane. Both VEGF and MMP-9 overexpression contribute to vascular invasion and poor prognosis in HCC [27,28,29]. The nuclear factor kappa-light-chain-enhancer of activated B cells (NF-κB) family which consists of five subunits—p65 (RelA), RelB, c-Rel, p105/p50 (NF-κB1), and p100/52 (NF-κB2)—regulates immunity, inflammation, differentiation, and tumorigenesis. The classical NF-κB heterodimer (p50/p65) modulates cancer hallmarks such as cell proliferation, evasion of apoptosis, and metastasis via inducing expression of proliferating, anti-apoptotic, and metastasis-associated proteins encoded by NF-κB target oncogenes [30,31]. Previous studies have demonstrated that anti-apoptotic proteins, proliferative and metastasis-associated proteins (MMP-9 and VEGF), and cell invasion were reduced by specific inhibition of NF-κB activation in HCC, lung, and glioma cells [27,32,33,34,35]. In the results presented, fluoxetine significantly reduces NF-κB activation, protein levels of Cyclin-D1, VEGF, and MMP-9 and inhibits cell migration/invasion in SK-Hep1 cells.

Mitogen-activated protein kinase/extracellular-signal-regulated kinase (MAPK/ERK) is the critical activator for tumor progression through upregulation of downstream kinases and transcription factors [36]. High expression of phosphorylated ERK (pERK) as the prognostic factor is linked to aggressive tumor behavior and poor overall survival in HCC [37]. Fluoxetine has also been shown to trigger apoptosis by inhibiting phosphorylation of ERK in Hep3B cells [13]. Previous studies indicated that PD98059, an ERK inhibitor, diminishes NF-κB activation and sorafenib, regorafenib, anti-HCC drugs, inhibit NF-κB-modulated tumor progression via suppression of ERK activation in HCC in vitro and in vivo [4,32,38]. Tine et al., reported that 5 µM fluoxetine did not affect ERK phosphorylation, but induced cell invasion, NF-κB activation, and expression of metastasis-associated proteins (MMP-2 and -9) in HCC HepG2 cells [39]. However, 30 µM of fluoxetine may induce cytotoxicity and ERK dephosphorylation in HCC Hep3B cells [13]. In a previous study, we demonstrated that NF-κB modulated invasive potential was diminished with 40 µM fluoxetine treatment in non-small cell lung cancer in vitro [35]. Notably, in this study, we indicated that 10–40 µM fluoxetine significantly reduced NF-κB activation, and 30–40 µM fluoxetine markedly suppressed protein levels of pERK, Cyclin-D1, VEGF, MMP-9, and cell migration/invasion in SK-Hep1 cells.

In conclusion, fluoxetine not only induces apoptosis through extrinsic/intrinsic pathways but also inhibits ERK/NF-κB-modulated anti-apoptotic and metastatic activity in HCC SK-Hep1 and Hep3B cells in vitro. We suggest fluoxetine as a potential inhibitor of ERK/NF-κB signaling which may provide therapeutic benefits for the treatment of HCC.

## 4. Materials and Methods

### 4.1. Chemicals, Antibodies and Reagents

Fluoxetine, MTT (3-(4,5-Dimethylthiazol-2-yl)-2,5-Diphenyltetrazolium Bromide), RNase and dimethyl sulfoxide (DMSO) were obtained from Sigma Chemical Co. (St. Louis, MO, USA). Dulbecco’s Modified Eagle’s (DMEM), fetal bovine serum (FBS), L-glutamine and penicillin-streptomycin were purchased from GIBCO^®^/Invitrogen Life Technologies (Carlsbad, CA, USA). Primary antibodies against Matrix metalloproteinase-9 (MMP-9) (AB19016, 1:1000, rabbit, EMD Millipore, Billerica, MA, USA), vascular endothelial growth factor (VEGF) (ab1316, 1:1000, mouse, Abcam, Cambridge, England, UK), Cyclin-D1 (DCS-6, 1:1000, mouse, Thermo Fisher Scientific, Fremont, CA, USA), Phospho-extracellular-signal-regulated kinase (pERK1/2) (Thr202/Tyr204) (D13.14.4E, 1:1000, rabbit, Cell signaling Technology, Danvers, MA, USA), total ERK (tERK) (sc-154, 1:1000, rabbit, Santa Cruz, CA, USA), myeloid cell leukemia-1 (Mcl-1) (BV-438, 1:1000, rabbit, BioVision, Milpitas, CA, USA), cellular FLICE (FADD-like IL-1β-converting enzyme)-inhibitory protein (C-FLIP) (D16A8, 1:1000, rabbit, Cell signaling Technology), X-linked inhibitor of apoptosis protein (XIAP) (PA5-29253, 1:1000, rabbit, Thermo Fisher Scientific), Survivin (E-AB-14381, 1:1000, rabbit, Elabscience Biotechnology Inc, Houston, TX, USA), Fas (E-AB-40063, 1:1000, rabbit, Elabscience Biotechnology Inc), Fas ligand (FasL) (E-AB-31410, 1:1000, rabbit, Elabscience Biotechnology Inc), BAK (E-AB-30623, 1:1000, rabbit, Elabscience Biotechnology Inc), and β-actin (sc-47778, 1:1000, mouse, Santa Cruz) of western blotting were purchased from different companies as listed. Secondary antibodies, Peroxidase AffiniPure Goat Anti-Mouse IgG and Goat Anti-Rabbit IgG were purchased from Jackson ImmunoResearch (1:10000, West Grove, PA, USA).

### 4.2. Cell Culture

SK-Hep1 and Hep3B, human hepatocellular carcinoma, were both obtained from Professor Jing-Gung Chung’s laboratory and routinely tested for mycoplasma contamination [39]. Cells were grown in a humidified incubator with 5% CO_2_ at 37 °C and cultured in DMEM medium. DMEM was supplemented with 10% heat inactivated fetal bovine serum (FBS), 2 mM l-glutamine and antibiotics (100 units/mL penicillin, 100 µg/mL streptomycin).

### 4.3. Plasmid Transfection and Stable Clone Selection

1 × 10^6^ SK-Hep1 or Hep3B cells were seeded into a 10 cm dish overnight. Cells were transfected with NF-κB luciferase reporter vector (pNF-κB/*luc2*) (Promega, Madison, WI, USA) using jetPEI™ transfection agent (Illkirch, Bas-Rhin, France) as described [40,41]. After transfection, cells were grown in culture medium supplemented with 200 µg/mL of hygromycin B at 37 °C for two weeks. After hygromycin B selection, function of NF-κB reporter gene of survival clones were identified with an IVIS imaging system 200 (Xenogen, Alameda, CA, USA). SK-Hep1/*NF-κB-luc2* and Hep3B/*NF-κB-luc2* cells which SK-Hep1 or Hep3B cells constitutively express the function of NF-κB reporter gene were grown in culture medium containing 50 µg/mL of hygromycin B and used for NF-κB reporter gene assay.

### 4.4. 3-(4,5-Dimethylthiazol-2-yl)-2,5-Diphenyltetrazolium Bromide (MTT) Assay

Cell viability was performed by MTT assay. MTT powder was dissolved in phosphate-buffered saline (PBS) and prepared as 50 mg/mL stock. Sk-Hep1 or Hep3B cells (3 × 10^4^) were placed in a 96-well plate overnight and incubated with and without fluoxetine at the final concentration (10, 20, 30 and 40 µM) for 24 h or 48 h. Media were then refreshed and incubated with 100 µL MTT medium combo solution per well (MTT final concentration was 5 mg/mL) for 4 h. Finally, MTT solution was replaced by 100 µL DMSO per well and the absorbance ratio was measured by Tecan Sunrise Absorbance Microplate Reader (Tecan Group Ltd., Männedorf, Switzerland) at 570 nm [33].

### 4.5. NF-κB Reporter Gene Assay

SK-Hep1/*NF-κB-luc2* or Hep3B/*NF-κB-luc2* cells were seeded into 96-well plates at 3 × 10^4^/well overnight, and then treated with different concentrations (0, 10, 20, 30 and 40 µM) of fluoxetine for 48 h. After treatments, the cell medium in each well was replaced with 100 µL of 500 µM D-luciferin (Promega, Madison, WI, USA) and incubated for 15 min in the dark. The effect of fluoxetine on relative NF-κB activity was evaluated using an IVIS imaging system and corrected with cell viability as described [41].

### 4.6. Annexin V/ Propidium Iodide (PI) Staining

SK-Hep1 cells were seeded into 6-well plates with 5 × 10^5^/well overnight and then treated with different concentrations (0, 30, and 40 µM) of fluoxetine for 48 h. Annexin V-FITC Apoptosis Detection Kit (Vazyme Biotech Co. Lt, Nanjing, China) was used for apoptosis detection after fluoxetine treatment. After harvesting, cells were resuspended in 100 µL binding buffer to which was added 5 µL Annexin V-FITC (FL1) and 5 µL PI (FL2) staining solution for 15 min at 25 °C. Cells were washed and centrifuged with PBS at 1500 RPM for 5 min, resuspended in 400 µL binding buffer, and then measured immediately yielding a FL-1(Annexin V-fluorescein isothiocyanate (FITC)) vs FL-2 (PI) dot plot in flow cytometry (FACS) (BD Biosciences, FACS Calibur, San Jose, CA, USA) and analyzed with FlowJo software (version 7.6.1; FlowJo LLC, Ashland, OR, USA) [42].

### 4.7. Cell Cycle Analysis

SK-Hep1 cells (5 × 10^5^) were seeded into 6-well plates and treated with different concentrations (0, 30, and 40 µM) of fluoxetine for 48 h. Cells were trypsinized and collected. Cells were fixed in 70% ethanol for 24 h at −20 °C, rehydrated with ice-cold PBS, and then stained with 40 µg/mL propidium iodide (PI) reagent (Biovision) with 100 µg/mL RNase in the dark for 30 min. Cell cycle analysis was then performed with flow cytometry and analyzed with FlowJo software [43].

### 4.8. Measurement of Caspase-3 and -8 Activation

SK-Hep1 or Hep3B cells were seeded into 6-well plates at 5 × 10^5^ cells/well overnight and then treated with different concentrations (0, 30, and 40 µM) of fluoxetine for 48 h. Cells were stained with CaspGlow Fluorescein Active Caspase-3 or -8 Staining kit from BioVision (Milpitas, CA, USA) adhering to the manufacturer’s instructions. Briefly, cells were stained with active-caspase-3 (1 μL active caspase-3 FITC antibody in 300 μL PBS) or active-caspase-8 staining solution (1 μL caspase-8 PE antibody in 300 μL PBS) for 30 min in a humidified incubator with 5% CO_2_ at 37 °C, respectively. Cells were washed once and harvested with 200 μL wash buffer and centrifugation and then resuspended in 500 µl wash buffer for immediate FACS analysis [43]. Data were analyzed using FlowJo software.

### 4.9. Measurement of Mitochondria Membrane Potential (ΔΨ_m_)

SK-Hep1 or Hep3B cells were seeded into 6-well plates at 5 × 10^5^ cells/well overnight and then treated with different concentrations (0, 30, and 40 µM) of fluoxetine for 48 h. The changes of ΔΨ_m_ were investigated by 3,3′-Dihexyloxacarbocyanine Iodide (DiOC_6_) (Enzo Life Sciences, Farmingdale, NY, USA) staining. This process was followed by incubating cells with 4 µM DiOC_6_ working solution for 30 min at 37 °C. Then, the cells were resuspended in 500 µL PBS buffer and analyzed using flow cytometry [43,44].

### 4.10. Assessment of Fas-L and Fas Activation

Sk-Hep1 cells (5 × 10^5^) were seeded into 6-well plates at cells/well and treated with 0, 30 and 40 µM fluoxetine for 48 h. For detection of cell surface antigens, collected cells were treated with PE-conjugated anti-CD178 (FAS-L) antibody (BioLegend, Inc., San Diego, CA, USA) or FITC-conjugated anti-CD95 (Fas) antibody and incubated on ice for 15 min in the dark. Then, they were washed twice with 500 µL cell staining buffer and resuspended in 500 µL cell staining buffer for flow cytometry. Each assay included at least 10,000 gated events. The histograms obtained were analyzed using FlowJo software [45].

### 4.11. Western Blotting

3 × 10^6^ SK-Hep1 cells were placed in a 10-cm dish overnight and then treated with different concentrations (0, 30, and 40 µM) of fluoxetine for 48 h. Cells were collected by centrifugation and lysised with in a lysis buffer (50mM Tris-HCl (pH 8.0), 120mM NaCl, 0.5% Nonide P-40) for 30 min and the total protein was measured using a Pierce BCA Protein Assay Kit (Thermo Fisher Scientific). 40–60 µg of total protein from each treatment was separated by 10–12% sodium dodecyl sulfate polyacrylamide gel electrophoresis (SDS-PAGE), and then transferred to polyvinylidene difluoride (PVDF) membrane (FluoroTrans^®^ Pall Corporation, Port Washington, NY, USA) by electroblotting and blocked with 5% (*w*/*v*) non-fat milk in Tris buffered saline (TBS) containing 0.05% Tween-20. Blots were probed by using primary antibodies and β-actin, followed by secondary antibody and Immobilon Western Chemiluminescent HRP Substrate (Pierce, Rockford, IL, USA) for enhanced chemiluminescence as described previously [32,46]. The immuno-detected proteins from each sample were then revealed by the MultiGel-21 imaging system (TOP BIO CO., Taipei, Taiwan) and their band intensities were quantified using Image J (version 1.50, National Institutes of Health, Bethesda, MD, USA). Quantification data were normalized by β-actin expression and averaged over three repeated experiments.

### 4.12. Invasion and Migration Assay

Sk-Hep1 or Hep3B cells (3 × 10^6^ cells in a 10-cm dish) were pretreated without or with 30 and 40 µM of fluoxetine for 48 h and harvested for Transwell assay. The invasion or migration of Sk-Hep1 and Hep3B cells was investigated using a 8 µm pore size Transwell assay (BD Biosciences, Franklin Lakes, NJ, USA) as described previously [27]. The upper chamber of the 24-Transwell plate was covered with (invasion) or without (migration) 0.5% Matrigel. 1 × 10^6^ cells were placed in the upper chamber of a 24-Transwell plate in DMEM medium containing 1% FBS. The cells invade or migrate from the upper chamber containing 1% FBS to the lower chamber, containing 10% FBS in DMEM media for incubation for 48 h. At the end of incubation, on the underside of the membrane, invaded cells were fixed with 4% formaldehyde in PBS and stained with 3% crystal violate, and air-dried for 15 min. Then, the cell number was photographed under a light a microscope (Nikon ECLIPSE Ti-U) at ×100 and quantified by ImageJ software [27].

### 4.13. Statistical Analysis

The results are presented as means ± S.D. from at least three experiments and the differences between fluoxetine-treated and control groups were measured by Student’s t-test. If *p*-values were <0.05, differences were considered statistically significant. Excel 2017 (Microsoft, Redmond, WA, USA) was used for statistical analyses in this study.

## 5. Conclusions

In this study, we suggested that fluoxetine induced apoptosis through extrinsic/intrinsic pathways. Moreover, fluoxetine may also decline ERK/NF-κB-modulated anti-apoptotic and invasive potential in two types of HCC cells (SK-Hep1 and Hep3B) in vitro.
